# Histology-based homogenization analysis of soft tissue: application to prostate cancer

**DOI:** 10.1098/rsif.2017.0088

**Published:** 2017-04-12

**Authors:** Javier Palacio-Torralba, Daniel W. Good, S. Alan McNeill, Robert L. Reuben, Yuhang Chen

**Affiliations:** 1 Institute of Mechanical, Process and Energy Engineering, School of Engineering and Physical Sciences, Heriot-Watt University, Edinburgh EH14 4AS, UK; 2 Edinburgh Urological Cancer Group, Division of Pathology Laboratories, Institute of Genetics and Molecular Medicine, University of Edinburgh, Western General Hospital, Crewe Road South, Edinburgh EH4 2XU, UK; 3 Department of Urology, NHS Lothian, Western General Hospital, Crewe Road South, Edinburgh EH4 2XU, UK

**Keywords:** homogenization, heterogeneity, tissue mechanics, cancer diagnosis, prostate cancer

## Abstract

It is well known that the changes in tissue microstructure associated with certain pathophysiological conditions can influence its mechanical properties. Quantitatively relating the tissue microstructure to the macroscopic mechanical properties could lead to significant improvements in clinical diagnosis, especially when the mechanical properties of the tissue are used as diagnostic indices such as in digital rectal examination and elastography. In this study, a novel method of imposing periodic boundary conditions in non-periodic finite-element meshes is presented. This method is used to develop quantitative relationships between tissue microstructure and its apparent mechanical properties for benign and malignant tissue at various length scales. Finally, the inter-patient variation in the tissue properties is also investigated. Results show significant changes in the statistical distribution of the mechanical properties at different length scales. More importantly the loss of the normal differentiation of glandular structure of cancerous tissue has been demonstrated to lead to changes in mechanical properties and anisotropy. The proposed methodology is not limited to a particular tissue or material and the example used could help better understand how changes in the tissue microstructure caused by pathological conditions influence the mechanical properties, ultimately leading to more sensitive and accurate diagnostic technologies.

## 1. Introduction

Biological tissues are often heterogeneous and hierarchical materials with complex underlying microstructures, which determine the apparent mechanical behaviour at the macroscopic level [1,2]. Such tissue microstructures can develop under certain pathophysiological conditions which may manifest themselves at the macroscopic level in such forms as lumps, inflammation and surface roughness and can consequently lead to changes in the mechanical properties. For example, the changes in soft tissue mechanical properties associated with breast [3], prostate [4], thyroid gland [5] and skin cancer [6] have been extensively investigated. Multiple sclerosis and aging have also been reported to influence the mechanical properties of brain tissue [7,8]. Although such variations in mechanical properties have been used for diagnosis for centuries, e.g. by palpation, in recent years the need for enhanced quantitative diagnostic procedures has emerged to complement and reduce the amount of biopsy and other non-innocuous procedures such as CT/MRI scans. As a result, various diagnostic techniques that exploit such changes in the mechanical behaviour of tissue have been developed. Elastography, which relies on the changes in the speed of propagation of elastic waves in media due to changes in stiffness, has been widely used in clinical diagnosis of liver tumours [9], prostate cancer [10] and atherosclerotic plaque [11]. Different palpation devices [12–14] and strategies [15,16] to detect variations in the mechanical properties have also been reported in the literature. It should be noted that most studies so far aimed at detecting conditions (e.g. malignant) at the tissue scale, rarely considered the microstructural cause of changes in mechanical properties. A model that takes into account the heterogeneous and hierarchical nature of tissue and its microstructural changes due to patho-physiological conditions could, when matched to multiscale mechanical measurements, deliver a quantitative relationship between the changes in tissue microstructure and macroscale mechanical properties. Such a model would allow the effects of intra-patient and inter-patient differences as well as pathological conditions to be taken into account, and would further develop such diagnostic techniques as elastography [17] and instrumented palpation [18].

Thus, quantifying the relationship between the mechanical behaviour of tissue and its underlying microstructures is a key step towards the ultimate aim of quantitative diagnosis and tissue quality assessment. This approach, also referred to as upscaling (e.g. homogenization, by deriving the macroscopic properties from underlying microstructures consisting of multiple constituents), has certain advantages:

— for the purpose of modelling, it significantly reduces the computational cost as the upscaled properties represent the apparent behaviour from the lower length scales; and
— from the clinical point of view, it enables more effective diagnosis based on the apparent mechanical properties of tissue as a primary diagnosis method.

Various methods have been proposed to estimate the effective properties of heterogeneous materials. It is often assumed that a representative volume element (RVE) can be found, which implies the existence of RVE periodicity in the sample being analysed or that the sample can be considered as an infinite volume containing a large set of RVEs [19]. Using the variational principle, Hashin & Shtrikman [20] proposed theoretical bounds for the effective mechanical properties independently of the topology of the inclusion. Despite the efficiency of analytical methods in estimating the effective properties of heterogeneous and porous or composite materials [21,22], numerical methods are more suited for nonlinear problems, for instance, when contact occurs within the sample being analysed [23]. It should be noted here that the mechanical properties of tissue may vary at different length scales. It is therefore crucial to examine the influence of the RVE size in the tissue mechanical assessment and the derived apparent properties.

Due to the heterogeneity and patient specificity in tissue microstructure, the mechanical properties of tissue often present a high degree of variability, although different, across all length scales. In fact, experimental characterization has shown that there may not even be a clear division between the mechanical properties of benign and malignant tissues, e.g. in prostate [13], for all individuals, from place to place within an individual, perhaps even from time to time at a given place in a given individual, depending on other physiological variables such as blood pressure and blood volume. Instead, a transition with varying mechanical properties is often found, which may add uncertainty to diagnosis. Furthermore, the inter-patient differences could significantly complicate diagnosis by adding another level of uncertainty [24]. Therefore, it is essential to characterize the statistical variations of the tissue properties and microstructures to enable quantitative diagnosis.

This study aims to establish a computational framework quantifying the relationship between the mechanical behaviour of tissue and its microstructural heterogeneity in histology caused by pathological conditions and also inter-patient variability. Prostate tissue samples are used to demonstrate the feasibility of the proposed methodology, although the framework is not restricted to any particular type of tissue. The effects of region of interest (ROI) size on the apparent homogenized properties and inter-patient variability will be investigated to assess the multi-scale effect. The proposed approach in this study, unlike (but complimentary to) those reported in the literature based on the principle of constitutive modelling (e.g. in the applications of arterial wall mechanics [22] and abdominal muscle [23]), focuses on the structural heterogeneities in histology caused by different pathophysiological conditions (e.g. benign and malignant prostate tissues) and their impact on the resulting apparent properties. The ultimate aim is to facilitate quantitative diagnosis by providing a means by which detailed microstructural and mechanical measurements can be used to develop structure–property relationships in tissue, which overcome the heterogeneity and patient-specificity issues mentioned above. The potential impact is an objective diagnosis of a wide variety of diseases (such as breast cancer, prostate cancer or liver fibrosis) using non-invasive mechanical diagnostic techniques such as palpation or elastography.

## 2. Material and methods

In this section the methodology is described as follows: firstly, the method used to convert images of tissue histology into ROIs is presented; secondly the formulation of homogenization used to estimate the apparent properties of tissue samples is described. Finally, the computational method for imposing periodic boundary conditions on samples with non-periodic boundary FE meshes is demonstrated.

### 2.1. Tissue samples from histology

**Figure 1.**
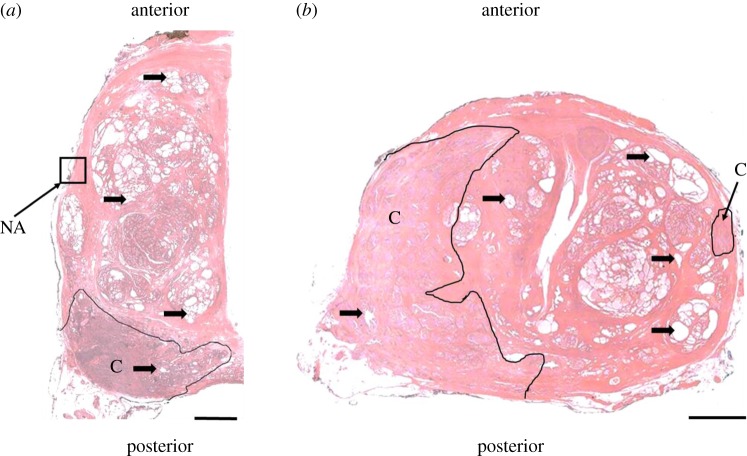
Histological slides stained with haematoxylin and eosin (H&E) from two patients. The black arrows illustrate examples of prostate acini. (*a*) Sample slide from Patient 1. The cancerous nodule is located at the posterior left side and marked with ‘C’. NA indicates an example of a region that would not be used as an ROI since it contains background of the slide. (*b*) Sample slide from Patient 2. This patient has two cancerous nodules located at the left and right sides of the prostate. Scale bars, 5 mm. (Online version in colour.)

Two patients only were selected for this study in order to enable an intensive investigation of the influences of inter-patient difference and various pathological conditions (both benign and malignant) on the derived mechanical properties of prostatic tissue. Both patients had undergone radical prostatectomy, followed by the preparation of histopathological slides of transverse sections. The pathological analysis of Patient 1's prostate revealed acinar adenocarcinoma with Gleason 3 + 4 and a prostate volume of 158 cm^3^, while Patient 2 was diagnosed with a more aggressive acinar adenocarcinoma with a Gleason 4 + 3 grade (Gleason grading is a histological grading method based on analysis of the tissue microstructure that helps evaluate the prognosis of a patient with prostate cancer [25]). Figure 1 shows one histological slide from each patient with standard H&E staining. The black outlines were drawn by the pathologist to highlight the boundary between the benign and malignant tissue.

To create FE models, the images were reconstructed using Scan-IP (Simpleware Ltd, Exeter, UK). First, the images were converted to greyscale and then a threshold of 180 in greyscale, (where 0 is black and 255 is white), was used to distinguish between the phases of solid tissue and interstitial fluid. To remove noise from the image a Gaussian recursive filter and island removal filter were used.

As mentioned above, the use of periodic boundary conditions implies the existence of periodicity across RVEs, which may not strictly be applicable to most biological tissues [19]. For this reason, the image samples used in the following analysis will be referred to as ‘regions of interest’ (ROIs). The entire histological models were then divided into square ROIs of three different side sizes, i.e. 0.67 mm (50 × 50 pixels), 1.34 mm (100 × 100 pixels) and 2.68 mm (200 × 200 pixels), with the aim of determining whether there is (within this range, at least) a characteristic size (i.e. length scale) at which the mechanical properties of cancerous and non-cancerous tissue are significantly different, thus providing best sensitivity for diagnosis. It should be noted here that the regions that contained either the white background of the histological slide, both tissue types, or the region close to urethra were discarded. As a result, for Patient 1, a total of 1274 non-cancerous and 290 cancerous samples for ROI size of 0.67 mm, 383 non-cancerous and 59 cancerous samples for size 1.34 mm, 89 non-cancerous and 11 cancerous samples for size 2.68 mm were obtained. For Patient 2 the numbers of samples are 1966 and 997 (0.67 mm), 443 and 217 (1.34 mm) and 84 and 39 (2.68 mm), for non-cancer and cancer, respectively. A summary of the resulting numbers of ROIs in each section at each scale is given in table 1.

**Table 1.** Summary of the number of samples and sizes of ROIs used in the study.

	0.67 mm (50 × 50)		1.34 mm (100 × 100)		2.68 mm (200 × 200)	
ROI size	non-cancer	cancer	non-cancer	cancer	non-cancer	cancer
Patient 1	1274	290	383	59	89	11
Patient 2	1966	997	443	217	84	39

The solid phase of each ROI considered for the analysis was modelled as a linear elastic material with an elastic modulus of 17 kPa [26] and a Poisson's ratio of 0.3. For the fluid phase (which in this work is considered as Newtonian and, due the quasi-static nature of the mechanical analysis, incompressible) a softer and nearly incompressible solid (*ν* = 0.499) with a bulk modulus of 2 GPa was considered [27]. For simplicity, small strains were considered in the histology-based modelling.

### 2.2. Formulation of numerical homogenization

Numerical homogenization is used here to obtain the effective properties of the chosen tissue samples (i.e. ROIs from the histological image). A number of different boundary conditions have been proposed in the literature for the numerical homogenization scheme. Kinematic uniform boundary conditions (KUBC) consist of a set of prescribed displacement fields and often provide upper bounds for the apparent stiffness, whereas static uniform boundary conditions (SUBC) often result in a lower bound by imposing a set of stress fields instead [28]. Obtaining upper and lower bounds is an interesting preliminary exercise, however it only really applies in extreme scenarios. Periodic boundary conditions (PBCs) which constrain the displacement at the boundaries to be periodic with imposed strain fields in the vertical, horizontal and shear components, provide intermediate apparent properties and are often used to estimate the apparent properties of materials and biological tissue with complex microstructures [19,29,30] and are hence adopted in this study.

To impose periodicity at the boundaries of an ROI, meshes with matching nodes at the corresponding sides are often required. However, this may require extremely fine meshes at the boundary especially when the geometry is reconstructed from medical images with complex topologies. Different methods that use, among others, Lagrange polynomials and spline interpolations have been reported in the literature to overcome such drawbacks [31,32]. However, they have certain limitations in the shape of the ROI (e.g. triangular), or in their compatibility with finite-element codes where accessing the stiffness matrix is difficult, sometimes impossible. Additionally they may significantly increase the number of variables in the finite-element analysis and consequently the computational cost. In this study a novel methodology that imposes the PBC using a set of control points periodically distributed over the ROI boundaries is proposed and validated. In particular the displacement at those points is calculated using the interpolation functions of and integrated in the finite-element analysis. A detailed formulation of the proposed methodology will be given in appendix A and its verification using benchmark structures will be shown in appendix B.

## 3. Results and discussion

### 3.1. Effect of region of interest size

**Figure 2.**
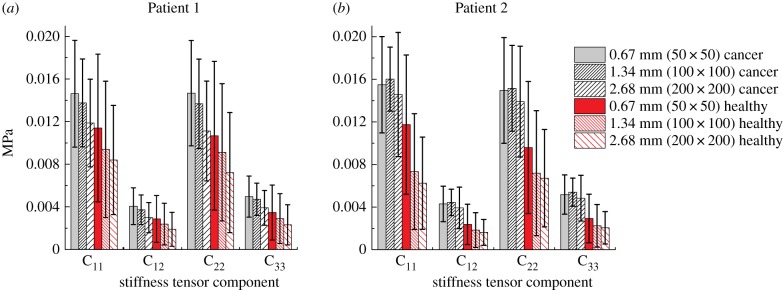
Average mechanical properties of the non-cancerous and cancerous ROIs from Patient 1 (*a*) and Patient 2 (*b*) and their standard deviations when the ROI size varies. (Online version in colour.)

The aim of this section is to investigate the influence of ROI size in the effective properties of tissue samples. Figure 2 shows the components of the apparent stiffness tensor for the two categories of tissue, i.e. cancerous and non-cancerous, for both patients, when three different ROI sizes are used (results are also listed in table 2). It should be noted that the average stiffness values of non-cancerous tissues are lower than those of cancerous tissue for all cases considered, which is consistent with what has been reported in experimental studies [3,18,26]. Table 3 shows the results of the Mann–Whitney *U*-test of such data, where statistically significant differences in apparent properties between non-cancerous and cancerous tissues can be observed in all cases, e.g. as measured by C_11_. Nevertheless, the statistical variation in the effective properties of non-cancerous and cancerous samples is significant and it is significantly larger in the non-cancerous samples than in cancerous ones. Such results could be attributed to a higher degree of heterogeneity in the non-cancerous tissue where acini, small, fluid-filled cavities surrounded by epithelial cells, are present in various sizes compared to cancerous tissue where acinar size is smaller and relatively less dispersed. This highlights the fact that considering just one component (or an insufficient number) from the elasticity tensor may lead to some inaccuracy in diagnosis.

**Table 2.** C_11_ (in kPa) of the average stiffness tensor and its standard deviation using different ROI sizes for Patient 1 and Patient 2. Terms C_31_, C_32_, C_13_ and C_23_ are at least three orders of magnitude lower therefore their data are not shown.

	cancer (0.67 mm)	cancer (1.34 mm)	cancer (2.68 mm)	non-cancer (0.67 mm)	non-cancer (1.34 mm)	non-cancer (2.68 mm)
C_11/Patient1_	14.6 ± 5.01	13.8 ± 4.12	11.9 ± 4.11	11.4 ± 6.93	9.40 ± 6.4	8.40 ± 5.12
C_11/Patient2_	15.5 ± 4.52	16.0 ± 3.0	14.6 ± 5.83	11.7 ± 6.54	7.33 ± 5.44	6.25 ± 4.33
C_12/Patient1_	4.07 ± 1.72	3.72 ± 1.39	2.98 ± 1.44	3.08 ± 2.22	2.38 ± 1.96	1.87 ± 1.61
C_12/Patient2_	4.29 ± 1.67	4.44 ± 1.25	3.89 ± 1.97	3.37 ± 2.06	1.85 ± 1.63	1.65 ± 1.21
C_22/Patient1_	14.7 ± 4.95	13.7 ± 4.2	11.1 ± 4.69	10.7 ± 6.98	9.12 ± 6.44	7.22 ± 5.64
C_22/Patient2_	15.0 ± 4.86	15.1 ± 4.03	13.9 ± 5.2	9.59 ± 6.2	7.19 ± 5.87	6.71 ± 4.57
C_33/Patient1_	4.96 ± 1.93	4.71 ± 1.51	3.91 ± 1.63	3.47 ± 2.58	2.90 ± 2.34	2.31 ± 1.89
C_33/Patient2_	5.18 ± 1.84	5.40 ± 1.32	4.84 ± 2.14	2.93 ± 2.29	2.25 ± 2.0	2.05 ± 1.52

**Table 3.** Summary the results of the Mann–Whitney *U*-test for equal means of C_11_, as an example, between non-cancerous and cancerous tissues, of each patient, when different ROI sizes are used. *H* = 1 means the null hypothesis, i.e. equal means in both cases, is rejected, indicating a statistically significant difference in apparent properties between non-cancerous and cancerous tissues.

case	*H* value	*p*-value
Patient 1/ROI size: 0.67 mm	1	3.1 × 10^−31^
Patient 1/ROI size: 1.34 mm	1	8.6 × 10^−18^
Patient 1/ROI size: 2.68 mm	1	1.3 × 10^−4^
Patient 2/ROI size: 0.67 mm	1	9.3 × 10^−30^
Patient 2/ROI size: 1.34 mm	1	2.3 × 10^−14^
Patient 2/ROI size: 2.68 mm	1	6.3 × 10^−4^

In the case of Patient 1 the average values of components in the stiffness tensor tend to reduce when the size of ROI is greater than 1.34 mm. Such reduction is caused by a greater number of acini being present in some ROIs. Patient 2 shows a similar trend although the difference between the distribution of the stiffness components of ROIs with sizes of 0.67 mm and the 1.34 mm is smaller. As mentioned above, this is due to the amount and size distribution of acini which can also be observed in figure 1, where a greater number of smaller acini in Patient 2 can be observed than in Patient 1. Interestingly, there is clear evidence of patient specificity in predicted mechanical properties of tissue samples at the larger scales. The average apparent stiffness of cancerous tissue observed in Patient 2 is in general higher than Patient 1 (measured by C_11_, C_22_ and C_33_ as shown in table 2), which suggests a lower amount of acini and potentially a more aggressive tumour in Patient 2. Table 4 shows the results of the Mann–Whitney *U*-test. At the smallest ROI size (i.e. 0.67 mm) the inter-patient difference is relatively small in the non-cancerous tissue. However, in all other cases (including non-cancerous samples with larger ROI sizes), there is significant inter-patient variability, e.g. as measured by C_11_. This will be discussed further in the next section.

**Table 4.** Summary the results of the Mann–Whitney *U*-test for equal means of C_11_, as an example, between two patients, in each tissue condition and ROI size used. *H* = 0: no statistically significant difference; *H* = 1: statistically significant difference.

case	*H*	*p*-value
non-cancerous/ROI size: 0.67 mm	0	0.2126
non-cancerous/ROI size: 1.34 mm	1	0.0045
non-cancerous/ROI size: 2.68 mm	1	0.0071
cancer/ROI size: 0.67 mm	1	7.8 × 10^−9^
cancer/ROI size: 1.34 mm	1	5.2 × 10^−6^
cancer/ROI size: 2.68 mm	1	0.0122

Most of the tissue samples considered in this study present a certain degree of anisotropy with a lower stiffness along the anteroposterior axis (i.e. C_22_). Such difference is rather obvious in the case of the non-cancerous tissue, particularly for samples in size of 0.67 mm. In fact, the literature shows certain directionality in prostatic tissue [33], which suggests the presence of anisotropy in non-cancerous tissue samples. However, in the cases of cancerous tissue, there appears to be significantly less anisotropy since the values of the diagonal components C_11_ and C_22_ are similar. This would indicate that the stroma is randomly oriented and has little directionality in cancer. Such disorganized tissue microstructure could be directly linked to the Gleason scoring system of the tissue sample with increased stroma content between acini, which further reduce its average size and density in cancerous tissue. In fact, the lack of recognizable glands (with microstructural features becoming irregular) is indeed a sign of a more malignant carcinoma corresponding to a higher Gleason score.

**Figure 3.**
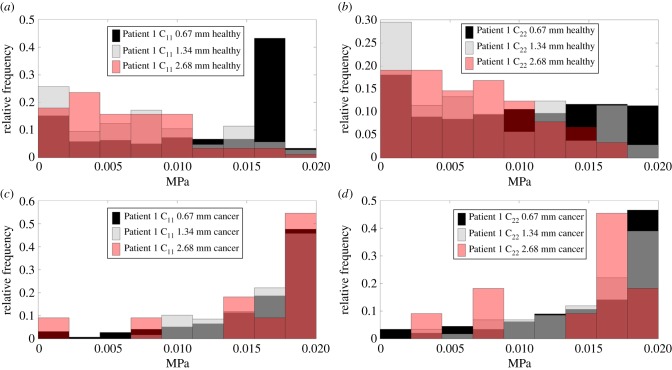
Comparison of the relative frequency of the magnitude of components C_11_ and C_22_ of the stiffness tensor for different ROI sizes for both non-cancerous and cancerous tissue for Patient 1. (Online version in colour.)

Figure 3 shows the relative frequency (i.e. the normalized number of times that the apparent value of the stiffness component appears within a certain range) of the magnitude of components C_11_, C_22_ and C_33_ of tissue samples from Patient 1, when different ROI sizes are considered. Results derived from Patient 2 present similar trends (data shown and discussed in the next section for the purpose of patient specificity). By comparing the statistical distributions between figure 3*a,b* to figure 3*c,d* it can be seen, as already illustrated in figure 2, the stiffness of cancerous tissue tends to be higher than that of non-cancerous tissue. It is important to note that the statistical distributions of the mechanical properties vary when the ROI size changes. For C_11_ in non-cancerous tissue, figure 2*a* shows a peak around 15 kPa when an ROI size of 0.67 mm is used, which is not present when greater ROI sizes are used and is, in fact, related to areas with lower density of acini. By comparison, the distribution of C_22_ is rather uniform as observed in figure 3*b*. This result implies that, at the ROI scale of 0.67 mm, the tissue presents a certain degree of anisotropy. As shown in table 5 anisotropy can be observed in all cases using the smallest ROI size for both cancerous and non-cancerous samples in both patients, whereas there are no statistically significant differences between C_11_ and C_22_ in other cases. This finding is in line with what has been observed from figure 2. Results for the cancerous tissue are however significantly different. The distributions of mechanical properties are similar between cases using the ROI sizes of 0.67 mm and 1.34 mm, especially in the region of higher stiffness values as seen in figure 3*c,d*.

**Table 5.** Summary the results of the Mann–Whitney *U*-test for isotropy (i.e. C_11_ = C_22_), for each patient and tissue condition, when different ROI sizes are used. *H* = 0: no statistically significant difference (isotropy); *H* = 1: statistically significant difference (anisotropy).

Case	*H*	*p*-value
Patient 1/non-cancer/ROI size: 0.67 mm	1	1.70×10^−18^
Patient 1/non-cancer/ ROI size: 1.34 mm	0	0.6927
Patient 1/non-cancer/ ROI size: 2.68 mm	0	0.5203
Patient 2/non-cancer/ROI size: 0.67 mm	1	7.4951×10^−5^
Patient 2/non-cancer/ ROI size: 1.34 mm	0	0.5182
Patient 2/non-cancer/ ROI size: 2.68 mm	0	0.1463
Patient 1/cancer/ROI size: 0.67 mm	1	0.0413
Patient 1/cancer/ ROI size: 1.34 mm	0	0.2814
Patient 1/cancer/ ROI size: 2.68 mm	0	0.6458
Patient 2/cancer/ROI size: 0.67 mm	1	4.7686×10^−4^
Patient 2/cancer/ ROI size: 1.34 mm	0	0.9554
Patient 2/cancer/ ROI size: 2.68 mm	0	0.5894

There are two points that should be noted here. Firstly, for diagnostic purposes, the distinguishability between two tissue types, as shown in figure 3, varies at different length scales. This leads to the possibility of optimizing the ROI size in order to achieve optimal diagnostic sensitivity. It must be acknowledged that there is a limited number of samples when the largest ROI size (i.e. 2.68 mm) is considered (as shown in table 1), leading to some inaccuracy in the representation of the statistical distribution of the tissue properties. Therefore, for discussion of the inter-patient difference later in this study, the ROI size of 2.68 mm will not be considered. Secondly, the statistical distributions of tissue properties change significantly with varied length scale (i.e. ROI size), and therefore it may be impossible to strictly define a representative ROI in tissues with a high degree of heterogeneity, hence the adoption of the term ROI in this study.

### 3.2. Inter-patient difference

**Figure 4.**
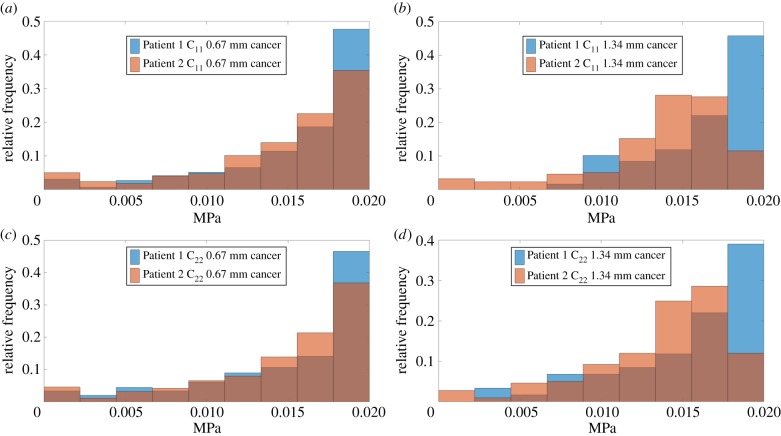
(*a*–*d*) Inter-patient comparison of the relative frequency of the apparent mechanical properties of ROI for different sizes when only cancerous tissue is considered. (Online version in colour.)

**Figure 5.**
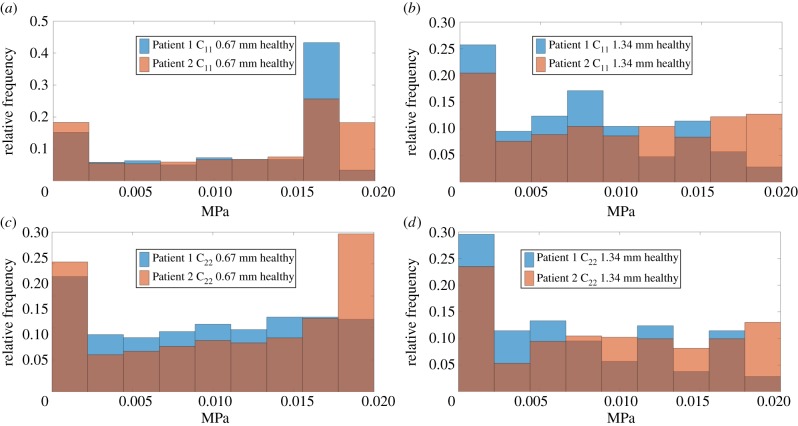
(*a*–*d*) Inter-patient comparison of the relative frequency of the apparent mechanical properties of ROI for different sizes when only non-cancerous tissue is considered. (Online version in colour.)

Figures 4 and 5 show the relative frequency of C_11_ and C_22_ of tissue samples from both patients, respectively, when the two different ROI sizes (i.e. 0.67 and 1.34 mm) are considered. The distribution of apparent stiffness is similar between C_11_ and C_22_ especially when the smaller ROI size is used and a similar trend is observed for the greater ROI size. Using C_11_ in cancerous tissue as an example, Patient 2, who had a more aggressive adenocarcinoma (Gleason 4 + 3, as opposed to Gleason 3 + 4 for Patient 1), has a wider range of stiffness values particularly towards the lower end of the stiffness as illustrated in figure 4*b*. This suggests a certain degree of heterogeneity caused by a less organized tissue microstructure (corresponding to less recognizable glands in the histological pattern). There is also a significant difference, particularly towards the higher stiffness areas, and this could be related to a lower proportion of glands present in Patient 2 which correlates well to the higher Gleason score diagnosed by the pathologist. Furthermore, in general, when the ROI size increases the differences between the distributions also increases.

**Figure 6.**
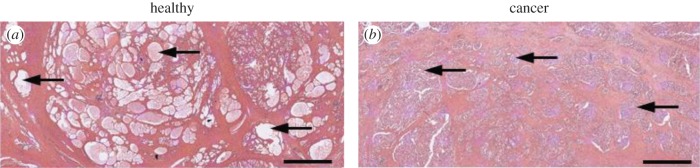
Comparison of the acini size and geometry between cancerous tissue (*a*) and non-cancerous tissue (*b*) shows that non-cancerous tissue contains larger and more regular/recognizable acini structures compared to the unrecognizable glandular structures in the cancerous tissue. Scale bars, 2 mm. (Online version in colour.)

It should be highlighted here that the length scale at which the mechanical characterization is carried out indeed affects the statistical distribution of the resulting tissue properties, which may have implications for the diagnostic information that can be extracted from such analysis. The noticeable inter-patient difference at a higher length scale (i.e. when ROI size of 1.34 mm is used), for example as shown in figure 4*b*, in comparison to the lower length scale (0.67 mm) in figure 4*a*, implies useful information about patient-specific features. These features include critical diagnostic information such as the presence of benign prostatic hyperplasia (BPH) or variations in the stroma density, and this could be further related to the acini structure and thus the grade of cancer [34] as illustrated in figure 6. On the other hand, measurement at a lower scale (using ROI size of 0.67 mm) could be more useful for a primary assessment of tissue quality where data are consistent with less difference induced by patient specificity (e.g. in figure 4*a,c*). These results suggest that, with a sufficiently large database of tissue properties, it would be possible to construct benchmark histograms for different types of pathologies, to which mechanical measurement of tissue could be directly mapped in order to assess the presence and grade of certain pathological conditions.

## 4. Concluding remarks

The aim of this study was to analyse how changes in the tissue microstructure (prostatic tissue as an example) caused by pathological conditions (e.g. presence of cancer) and inter-patient variation influence the apparent elasticity of a soft tissue considered to be made from solid and fluid tissue phases. Due to the complex geometries indicated by the histological images, a novel method that applies the periodicity across the ROI at the control points instead of FE nodes is proposed and later validated using a benchmark solution. Using this methodology, it is shown that the cancerous prostatic tissue presents statistically a lower degree of anisotropy than non-cancerous tissue and less statistical variation in tissue properties, although with significantly higher stiffness. The effects of the sample size have also been investigated and proven to be critical in assessing the statistical distribution of the tissue samples. Illustrative results suggest that tissue properties estimated from a higher length scale (i.e. when the ROI size is greater) could give insight into inter-patient variation, whereas results from the lower length scale (i.e. smaller ROI size) provide useful information for primary diagnosis.

It is important to remark here that the distribution of the mechanical properties obtained in this study for both non-cancerous and cancerous tissue are caused by the microstructural variation in the tissue samples, rather than by the changes in the intrinsic properties of the stromal tissue (which are assumed to be constant). Therefore, it can be concluded that the variation in tissue microstructure is an important factor affecting elasticity and anisotropy of tissue subject to the presence of cancer. The illustrative results suggest a strong link between tissue microstructure, mechanical properties and its pathological condition, which indicates some potential in using the mechanical properties of tissue as quantitative indices for clinical diagnosis.

The study presented here has some limitations as it stands. Firstly, both tissue phases are modelled as continuum solid materials. Although this assumption has been widely used in the literature, a more realistic fluid-structure interaction analysis may be required, particularly for the purpose of investigating the time-dependent behaviour in soft tissue. Secondly, the number of patients in this study is limited in order to carry out an intensive examination of these very complex structures. In fact, the same mechanical properties of tissue constituents were used for both patients since the aim of this work was to study the influence of the tissue microstructure rather than considering other patient-specific parameters such as those at cellular and sub-cellular levels, which may affect the degree of inter-patient variability presented in this study. This is expected to be included in potential future work, in addition to increasing the size of the dataset and including cancer at different grades/stages as well as other pathological conditions that can be further validated by instrumented palpation or tissue elastography. The ultimate aim would be to demonstrate if indices such as tissue elasticity and its heterogeneity and anisotropy can be quantitatively related to microstructural parameters such as average size and volume fraction of acini, and therefore become an adaptable tool for quantitative clinical diagnosis.

## Figures and Tables

**Figure 7.**
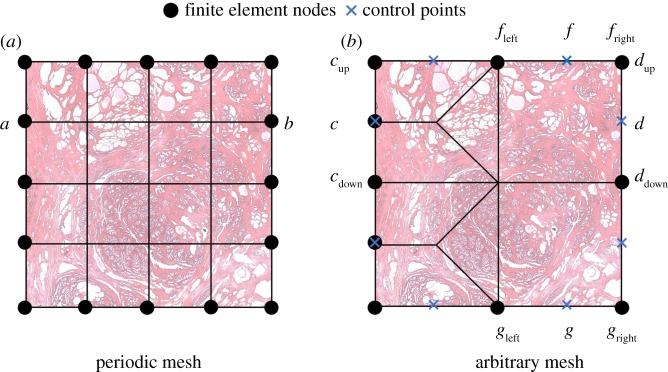
Schematic diagram of the proposed method of imposing PBC in a non-periodic mesh. (*a*) Example of a periodic mesh where nodes at all boundaries have a matching node at the opposite side. (*b*) A non-periodic mesh where some nodes (e.g. *c*) do not have a matching node at the opposite side. (Online version in colour.)

**Figure 8.**
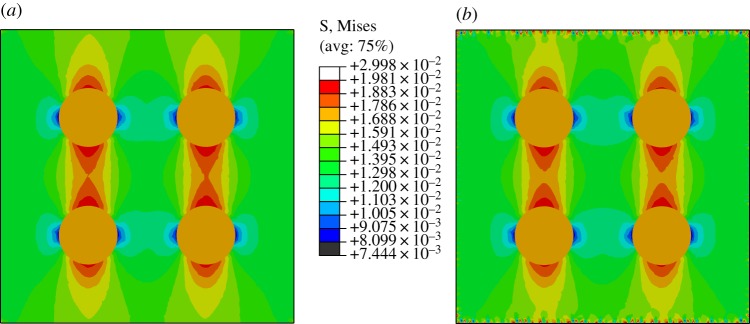
Distribution of Von Mises stress when the traditional method (*a*) and the new method (*b*) with 80 control points are used to impose periodic boundary conditions subject to a vertical tensile strain of 10%. In both cases the mesh contains 100 nodes at each side. It should be noted that the maximum Von Mises stress is present at the upper and lower boundaries and the area affected by those values is comparatively small to the rest of the RVE as shown in this figure. (Online version in colour.)

**Figure 9.**
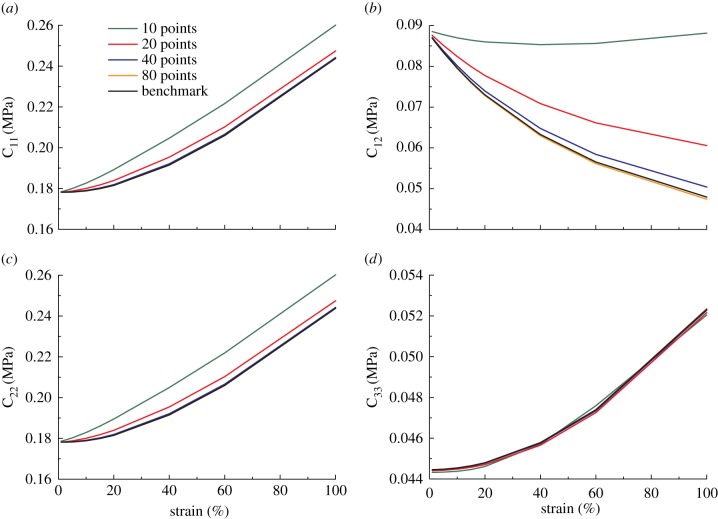
Convergence results towards the solutions from the traditional PBC method when the number of control points is increased. (*a–d*) show the results for the components C_11,_ C_12_, C_22_ and C_33_, respectively. (Online version in colour.)

## Data accessibility

Research data of this article are accessible through the DOI link (10.17861/136a1053-096c-450c-b57c-ae6eb1573793).

## Appendix A. Implementation of PBC on non-periodic FE meshes

Using the interpolation functions in a traditional scheme of finite-element analysis, it is possible to obtain variables such as displacement and strain at any location over the domain and, in particular, at the boundaries. To overcome the aforementioned challenge of the need for periodic FE nodes, it was decided in this study to consider a finite number of control points at each boundary, located periodically and, without loss of generality, distributed uniformly, which do not necessarily share locations with FE nodes. The constraints that impose the periodic displacements at the boundaries are considered at these control points instead of at the FE nodes (see [19,31,35] for details of the traditional method for imposing the PBC). Using the same interpolation functions as the ones used in the FE method, the displacements at the control points can be expressed as a combination (e.g. linear) of the displacements at FE nodes. Figure 7*a* shows a periodic mesh where every node in the boundary (e.g. node ‘*a*’) has a matching node at the opposite boundary (e.g. node ‘*b*’). In non-periodic meshes as shown in figure 7*b* that is not always the case as demonstrated by point ‘*c*’, which does not have a matching node at the opposite side. The following equations exemplify the displacement boundary conditions at nodes ‘*a*’ and ‘*b*’ of a periodic mesh when, for example, a vertical tensile strain is applied.

A 1

With the proposed methodology, in figure 2*b*, the constraints are imposed instead at control points ‘c’ and ‘d’

A 2

The displacement at the control point ‘*d*’ is not a variable of the FE problem, and therefore has to be converted back to the adjacent FE nodes, i.e. *d*_up_ and *d*_down_. In this particular case the same linear interpolation as the one used in the FE analysis is considered

A 3

where for this particular application *z* = *l*/2, and similarly for the *y* axis

A 4

where *l* is the distance between the two nodes.

In some cases, however, when the control point has no corresponding FE node at the other side, the displacements at both controls points need to be expressed as a function of the values at adjacent FE nodes, as demonstrated by the case of control points ‘*f*’ and ‘*g*’ in figure 7*b*

A 5

and

A 6

where  is the tensile strain applied along the *y* axis specified in this particular case. As a result of applying these imposed displacement constraints, the periodicity at the ROI boundaries is fulfilled at the control points instead of at the FE nodes.

**A.1. Verification of the proposed PBC method using control points**

To test the proposed methodology, an ROI often used as a benchmark structure [31], which consists of four circular inclusions embedded into a soft matrix, is employed. The benchmark model is meshed with 100 FE nodes each side. The test model is also meshed with 100 FE nodes on each boundary face but the constraints in periodicity are only considered at control points which are evenly distributed along the boundary. Three test strains are conducted: uniaxial tensile tests along the *x* and *y* axes and a shear test. In each test the average strain () and stress () are calculated across the ROI. To obtain the nine components of the stiffness tensor shown below, a set of equations below need to be satisfied:

A 7

and

A 8

where  and  denote the average stress and strain across the ROI, respectively, and  the effective stiffness tensor.

For the benchmark ROI structure, the influence of the number of control points in the estimated apparent stiffness is also analysed under large strain. Global uniaxial tensile strains along the *y* axis of up to 100% (i.e. 1%, 2%, 5%, 10%, 15%, 20%, 40%, 60% and 100%) are considered. The mechanical properties of the matrix and inclusions are modelled using a neo-Hookean model

A 9

where  is the first deviatoric strain invariant, *J* is the elastic volume ratio and *C* and *D* are material parameters. *C*_M_ = 0.02, *D*_M_ = 0.001 are assigned to the matrix material and *C*_1_ = 0.06, *D*_1_ = 0.001 to the inclusion, respectively. This leads to a behaviour when the material is perturbed from the zero strain configuration of approximately *ν* = 0.5, *E*_M_ = 120 kPa and *E*_I_ = 360 kPa. These values are arbitrary (although representative of soft tissue, e.g. brain and cartilage as two extreme examples [36,37]) and used to test the feasibility of the proposed methodology under the hypotheses of large strain and material quasi-incompressibility.

## Appendix B. Implementation and verification using the proposed PBC method

This section compares the apparent properties of an ROI obtained from the traditional and the proposed methods of implementing a PBC described above. Figure 8 shows the stress distribution for both cases subject to a tensile strain applied along the vertical direction. The main differences occur at the boundaries near the control points as a result of the forces required to enforce the periodicity. However, the influence of such stress concentrations on the estimated average properties is negligible as they only occur in very limited areas. Figure 9 shows the apparent properties obtained when various strains are applied and the convergence towards the solution from the traditional method when the number of control points increases. Due to the symmetries of the ROI its apparent properties can be considered to be orthotropic therefore only four components (i.e. C_11_, C_12_, C_22_, C_33_) in the stiffness tensor are shown. When a low number of control points (i.e. 10 at each side) is used the resulting values of the different components, especially in C_11_ and C_22_, are higher than those calculated from the benchmark method (using periodic mesh). With the increased number of control points, the results converge to the benchmark solution. C_11_ and C_22_ follow a similar trend and the error is within 5.2% when only 40 control points are used. The difference becomes negligible when more than 80 control points are present. C_12_, however, decays with increasing strain and needs more control points to achieve a similar level of accuracy. On the other hand, C_33_ is less sensitive to changes in the number of control points. Although increasing the number of control points would further reduce error as shown in figure 9, the number of control points cannot be increased without limit since there should be only one control point between the FE nodes otherwise the resulting equations could become over constrained. It can be seen that with 80 control points per side (compared to the 100 FE nodes), the accuracy of the method is not significantly compromised and results in a lower number of constraints and therefore a lower computational cost. More importantly, the proposed methodology requires no further mathematical manipulation of the stiffness matrix or load vector in the FE formulation which is critical, especially when the stiffness matrix and/or load vector are not accessible.
